# Characterization of Nano-Sized Features in Powder Bed Additively Manufactured Ti-6Al-4V Alloy

**DOI:** 10.3390/ma18133198

**Published:** 2025-07-07

**Authors:** Eyal Eshed, Amnon Shirizly

**Affiliations:** 1 Rafael Advanced Defense Systems Ltd., P.O. Box 2250, Haifa 3102102, Israel; 2 Faculty of Mechanical Engineering, Technion—Israel Institute of Technology, Haifa 3200003, Israel

**Keywords:** Ti-6Al-4V alloy, laser powder bed fusion, transmission electron microscopy, heat treatment

## Abstract

In this study, we delve into the intricate microstructural features of Ti-6Al-4V alloy additively manufactured and heat-treated at 800 °C for 4 h. Our in-depth analysis will enable us to gain a better understanding of the β-Ti precipitation process, its dependence on temperature, and its ultimate effect on the overall mechanical properties. As well as α-Ti martensite grains and β-Ti particles interspersed in the α-Ti grain boundaries, there is a third microstructural feature, overlooked by many researchers. This feature is observed as nano-sized particles homogeneously embedded inside the α-Ti laths. Using high-resolution transmission electron microscopy, we reveal that these nano-sized features do not constitute a different phase. Instead, they define isolated regions of α-Ti in its relaxed form, surrounded by the heavily strained form of the α-Ti phase. This phenomenon is a result of the “incomplete” precipitation of the β-Ti phase following the heat treatment stage. The straining of the α-Ti phase appears as a shift in the equilibrium atomic position.

## 1. Introduction

The microstructure of commercial Ti-6Al-4V alloy is highly dependent on the manufacturing process parameters and subsequent plastic working, as well as heat treatments. Consequently, numerous papers have been dedicated to this topic over the years [1,2,3,4,5,6,7,8,9,10,11]. Until the development of metal additive manufacturing (AM), titanium alloys were primarily produced either in wrought form by forging, or in cast form. The AM process for Ti-6Al-4V parts is unique in that an extremely fine metastable microstructure can be achieved in the as-built state, bearing similarities to those achieved through welding or quenching processes [12]. This characteristic significantly broadens the range of attainable microstructures and mechanical properties for AM Ti-6Al-4V parts, depending on subsequent heat treatments.

Ti-6Al-4V is part of the duplex α + β Ti alloy class. In the equilibrium state, and at relatively low temperatures, the microstructure of pure titanium consists of the α-Ti phase, which has an HCP (hexagonal close-packed) crystal structure. At elevated temperatures (above the β transus), β-Ti is the sole phase, with a BCC (body-centered cubic) crystal structure. The Ti-6Al-4V alloy contains several β stabilizing elements such as V and Fe, as well as an α stabilizing element: Al. The β stabilizing element V causes a reduction in the β transus temperature, relative to pure titanium, and consequently may lead to the retention of some fine β phase particles at room temperature, which would explain this alloy’s classification [1,2,3,4,11].

In LPBF, rapid solidification from the molten state causes the AM Ti-6Al-4V alloy to display non-equilibrium phases and morphologies. More specifically, there is little time for the β stabilizers to diffuse out of the newly formed α phase during the β-to-α transformation occurring in rapid solidification. As a result, the martensitic α′-Ti phase forms from prior β phase grains. This phase maintains the existing HCP structure of the α phase but contains increased concentrations of β stabilizers [1,5]. Therefore, the microstructure obtained for as-built Ti-6Al-4V only displays the martensite α′ phase in several lamellar or lath morphologies, often differentiated by their sizes, ranging from a few micrometers to tens of nanometers [5,6,7]. As indicated by Motyka [5], researchers often term these classes of laths primary, secondary, tertiary, and quartic α′ in order of descending size.

Upon rapid cooling, a single prior β-Ti grain will transform into several domains of fine α′ phase laths, such that each domain contains a single α′ variant according to 12 possible α′-β orientation relationships [13,14,15,16,17,18,19]. The fact that following the completion of this transformation, there are several neighboring domains means that there are also specific α′/α′ boundary types characterized by different angles and crystal lattice relations [13,14,15,16]. Additionally, changes in AM parameters can lead to different α′ variant selection during the β-α′ transformation, which may contribute to the overall anisotropic behavior, both mechanical and otherwise [17,18].

Heat treatments for Ti-6Al-4V can be divided into three categories: subtransus treatments, supersolvus treatments, and a mixture of both. In a subtransus treatment, the annealing tempering is below the β transus temperature of 995 °C. In a supersolvus treatment, the annealing temperature is above the β transus and a subsequent quenching step is added [3,6,10]. The subtransus treatment cannot alter the morphology of the prior β columnar structure, as opposed to the supersolvus treatment, which transforms the initial columnar structure of the prior β grains into equiaxed and half-equiaxed structures [6,10]. The quenching of Ti-6Al-4V after the supersolvus treatment results in the formation of new α′ particles, which can then be decomposed into α-Ti and β-Ti in a second subtransus treatment. This combination of the supersolvus and subtransus treatments constitutes the mixed treatment [6].

The most common heat treatment category for Ti-6Al-4V is subtransus. Depending on the subsequent subtransus heat treatment temperature, the as-built all-martensitic microstructure begins to transform as the α′ phase undergoes partial decomposition into separate lamellar α grains and β platelets [5,6,8]. In addition, when furnace cooling is allowed, the initial α′ phase is expected to completely decompose, leaving only Al-rich lamellar α grains and V-rich β platelets located at the α grain boundaries [6,8].

Several publications [2,5,6,20] have reported on the obtained microstructure in AM Ti-6Al-4V following a subtransus heat treatment at a moderate temperature of 800 °C, determining that only the lamellar α and particulate β phases exist. However, only Huang et al. [6] showed the existence of either nanoparticles or nano-sized globular features inside the α phase grains in scanning electron micrographs. Is this phenomenon specific to heat treatment at 800°C? When comparing with the obtainable microstructures at other tempering treatment temperatures, it is important to be cautious, primarily because there is probably a range of temperatures at which these nano-features may form. Secondly, this range depends on build parameters such as the laser power, scanning speed, layer thickness, etc., as well as heat treatment parameters such as the cooling regime. More specifically, compared with microstructures obtained in the literature for SLM Ti-6Al-4V products that were furnace-cooled, a tempering temperature below 700 °C yielded microstructures containing only α and α′ lath morphology in varying sizes, whereas at temperatures higher than 900 °C, the alloy contained a fully “mature” α + β microstructure, with no apparent nano-features [2,6,21].

Since these features also formed in AM Ti-6Al-4V under a similar heat treatment performed by the authors of this paper, they are the subject of the investigation presented herein. It is important to note that while α variant nanoparticles were reported in other α + β titanium alloys, such as 5553 [14] and TC21 [19], they had a characteristic needle shape and were embedded in a β–Ti matrix. The nano-features in this case appear to be unique to the AM process and to this specific heat treatment temperature. In light of that, it is essential that we accurately understand the nature and identity of these features and their impact on mechanical properties.

In this publication, we take a closer look at the general microstructure of a heat-treated additively manufactured Ti-6Al-4V alloy using optical microscopy, identify the nano-features inside the α-Ti phase lamellae through high-resolution scanning electron microscopy (HR-SEM), and characterize their crystal structure via high-resolution scanning/transmission electron microscopy (HR-S/TEM). Since only STEM mode was used in this publication, the verification of the crystal structure of each inspected phase will be presented as a high-angle annular dark field (HAADF) lattice image and its corresponding fast Fourier transform (FFT) in lieu of the selected area electron diffraction (SAED) pattern usually obtainable in TEM mode.

## 2. Experimental Procedure

The L-PBF (laser powder bed fusion) process was performed in an EOS-M280 3D-printer equipped (Krailling, Germany) with a 400 W fiber laser, using optimal parameters for supported and bulk structures as developed by the manufacturer. The layer thickness was 30 µm.

The raw material fed into the machine was a commercial Eckart TLS Ti-6Al-4V Grade 5 powder (Erlangen, Germany), with a chemical composition conforming to the specifications mentioned in [22]. The grain size distribution had the following parameters: D_10_ in the 20–26 µm range, D_50_ in the 34–40 µm range, and D_90_ in the 50–56 µm range.

Table 1 presents the typical chemical composition of the raw material powder and the final AM product, as obtained using mass spectrometry:

The resultant Ti-6Al-4V product was then subsequently heat-treated at 800 °C for 4 h under vacuum conditions using a Nabertherm vacuum furnace. The pressure throughout the treatment ranged between 10^−4^ and 10^−7^ torr, with the product being allowed to cool to room temperature inside the furnace.

A metallographic specimen for optical and HR-SEM investigation was prepared by cutting, “Bakelite“ mounting, and subsequent grinding and polishing. A final etching stage with Kroll’s reagent was performed via immersion for 10–15 s in order to enhance the phase contrast. SEM analysis was performed using a TESCAN-MIRA3 high-resolution field emission gun (FEG) SEM equipped (Brno, Czech Republic) with an Oxford EDS XMAX50N Aztec energy standard EDX (energy dispersive X-ray spectroscopy) microanalysis system (Buckinghamshire, UK).

A TEM specimen was prepared from the Ti-6Al-4V product using a Helios nano-lab G3 FEI dual-beam focused ion beam (FIB) high-resolution SEM microscope. High-resolution TEM analysis was conducted using an FEI Titan Themis G2 60-300 kV S/TEM equipped (Hillsboro, OR, USA) with HAADF and an EDX detector for chemical analysis.

## 3. Results and Discussion

**Figure 1:**
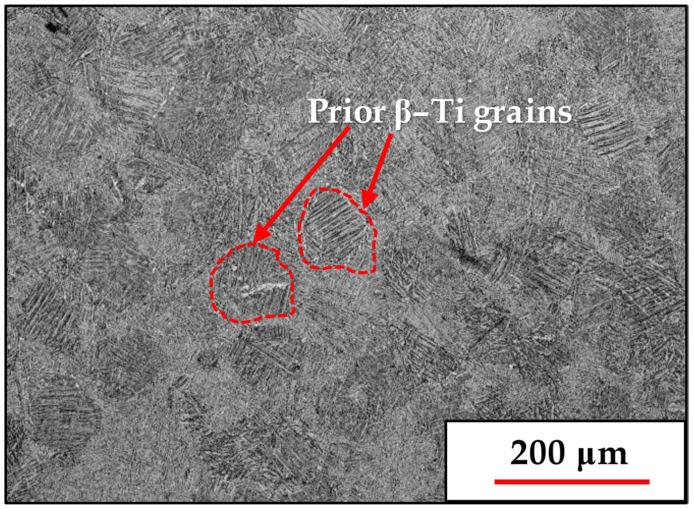
Optical micrograph of a transverse cross-section (parallel to the build direction) showing the typical prior β-Ti grains and the intricate lamellar α-Ti grain structure inside each prior β-Ti grain in the AM Ti-6Al-4V product following heat treatment at 800 °C.

The typical microstructure obtained for the AM Ti-6Al-4V products following heat treatment at 800 °C is shown in Figure 1:

The micrograph in Figure 1 shows the prior β-Ti grains, whose size of around 0.1 μm correlates with the hatching distance. Inside each prior β-Ti grain is an array of intersecting α-Ti lamellae whose spatial orientation relates to the parent β-Ti grain orientation in accordance with the 12 possible α variant formations during the β-to-α transformation [13,14,15,16].

**Figure 2:**
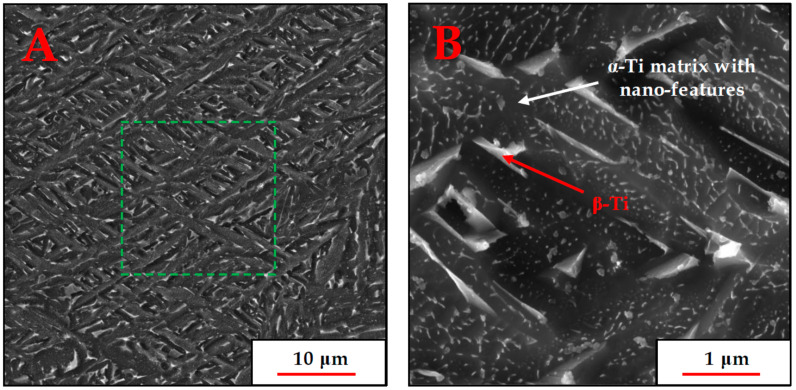
High-resolution SEM micrographs, in SE (secondary electron) detection mode, of the AM Ti-6Al-4V product following heat treatment at 800 °C, “Bakelite” mounting, polishing, and etching in Kroll’s reagent: (**A**) the distribution of the α-Ti grains and β-Ti particles; (**B**) the nano-sized features in each α-Ti grain. The dashed green square indicates the area used for image analysis to evaluate phase fraction.

SEM images demonstrating the phase morphology and distribution inside each prior grain are shown in Figure 2 in two magnifications.

Figure 2 shows the crisscrossing lath morphology of the α-Ti lamellae, as well as the presence of small β-Ti particles or platelets, situated along the α-Ti lamella boundary direction. The nano-sized features in the α-Ti grains appear to be ubiquitous and quite uniform in size, and to determine whether they constituted a different phase or some other phenomenon, a thorough high-resolution transmission electron microscopy (HR-TEM) investigation was performed.

**Figure 3:**
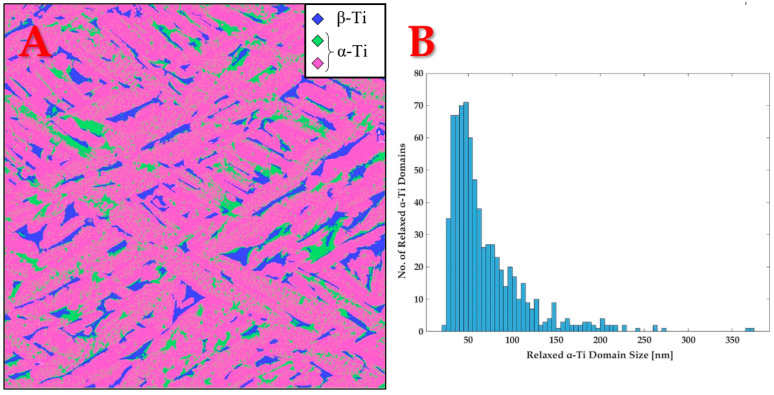
Microstructural quantification in AM Ti-6Al-4V following 800 °C heat treatment. (**A**) Image segmentation into phases and features: Dark blue represents β-Ti, light green represents the nano-scale features inside α-Ti, and pink represents α-Ti; (**B**) size distribution histogram of the nano-scale features, later proven to be the relaxed form of α-Ti. Bin size equals 5 nm.

To assess the volume fraction of each phase or feature in the microstructure, an image analysis of the dashed green region in Figure 2A was performed. The analysis was based on pixel brightness variation, as well as manual and automatic feature recognition in MATLAB version R2022b. Additionally, the size distribution of the nano-features was constructed, using the same image analysis technique on the micrograph in Figure 2B. It is important to emphasize that since the nano-scale features were not symmetrically shaped, their characteristic length was determined according to the definition of the maximum Feret diameter. This is the maximum measurable distance between two parallel tangent lines to the boundary curve of each feature. Both quantification results are provided in Figure 3:

Based on Figure 3A, the volume fraction of each feature was evaluated. The results are presented in Table 2:

From Figure 3B, the average and median characteristic length of the nano-scale features were determined to be 69.6 ± 43.9 nm and 54.8 nm, respectively.

**Figure 4:**
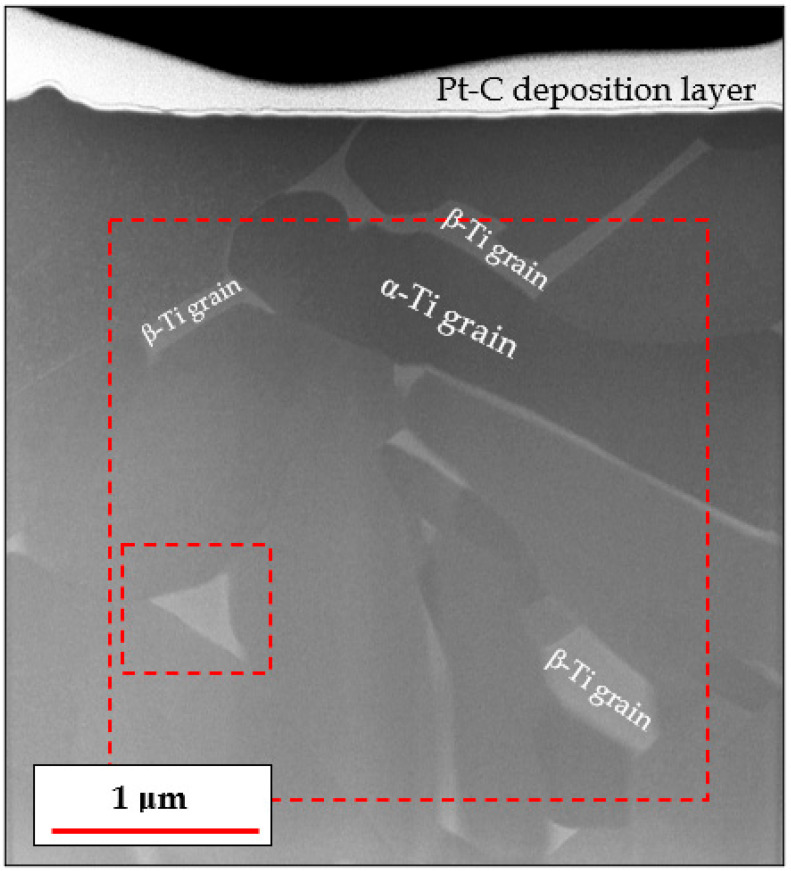
Phase distribution and grain structure of the heat-treated Ti-6Al-4V TEM specimen, as viewed using scanning transmission electron microscopy (STEM) in a neutral sample position (no sample tilting). The amorphous platinum–carbon layer is a feature of sample preparation by FIB and is not part of the investigated alloy. The dashed red squares represent areas where EDX measurements were obtained.

Figure 4 shows an overview of the TEM specimen in scanning transmission mode, prepared using a FIB microscope.

From Figure 4, it can be clearly observed that the brighter β-Ti particles are located at the α-Ti lamellar boundaries. This observation is to be expected since the heavier β-stabilizing elements, Fe and V, segregate away from the coarsening α-Ti lamellae during heat treatment and can therefore be found in high concentrations at the boundaries of each α-Ti lamella. This process culminates in the nucleation and growth of β-Ti particles in these α-Ti boundary regions.

The nano-sized features inside the α grains (as seen in Figure 2B) were not visible in STEM images, as shown in Figure 4. Furthermore, each α grain is seen as having a different brightness or grayscale intensity, based on how close or far it is, in terms of the spatial angle, from a low index zone axis relative to the neutral position of the TEM specimen, from which Figure 4 was obtained, i.e., where the specimen itself is perfectly perpendicular to the electron beam.

**Figure 5:**
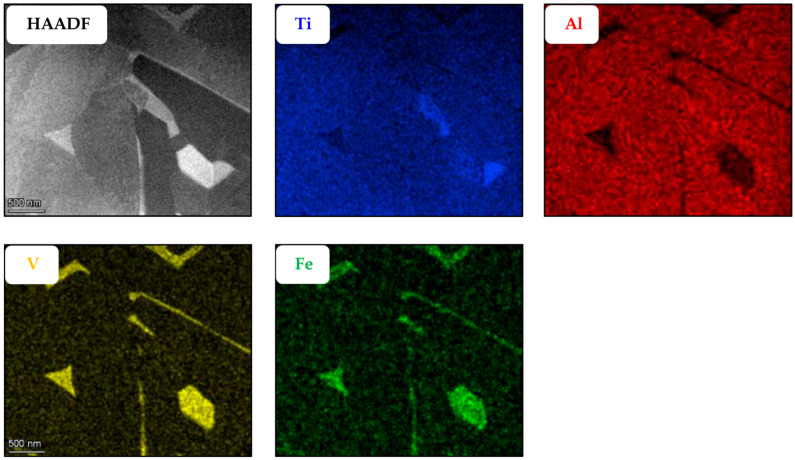
Low-magnification EDX mapping, showing the spatial distribution of the four main elements of the alloy, Ti, A, V, and Fe, in the α and β phases.

**Figure 6:**
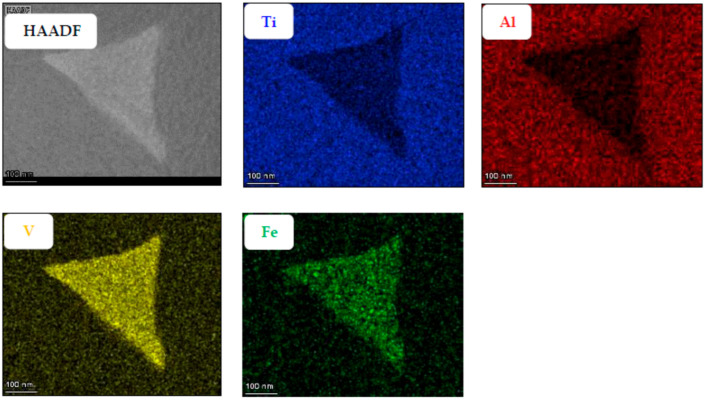
High-magnification EDX mapping, showing the spatial distribution of the four main elements of the alloy, Ti, A, V, and Fe, in the α/β interface regions, as well as inside α-Ti lamellae.

Another attempt at determining the location of the nano-features was performed via EDX. If these nano-features constituted a different phase, we would expect them to have a different chemical composition than the surrounding α-Ti lamella. EDX maps were obtained from two sparate regions of the specimen in order to visualize the difference in composition between the α and β phases and between the α phase and the nano-features embedded in it (Figure 5 and Figure 6 respectively).

From Figure 5 and Figure 6, it can be observed that among the alloying elements, Al is concentrated preferably in the α phase while V and Fe concentrate in the β phase, as expected. The nano-sized features, easily seen in the SEM micrograph presented in Figure 1 and previously believed by the authors to be the metastable martensitic phase-α′, are not noticeable in the EDX maps at all. If the particles were the martensite phase, their V and Fe content would be much higher than that of the surrounding α phase, similar to the composition of the β phase. Hence, the EDX maps negate the existence of such α′ nanoparticles inside the α phase.

**Figure 7:**
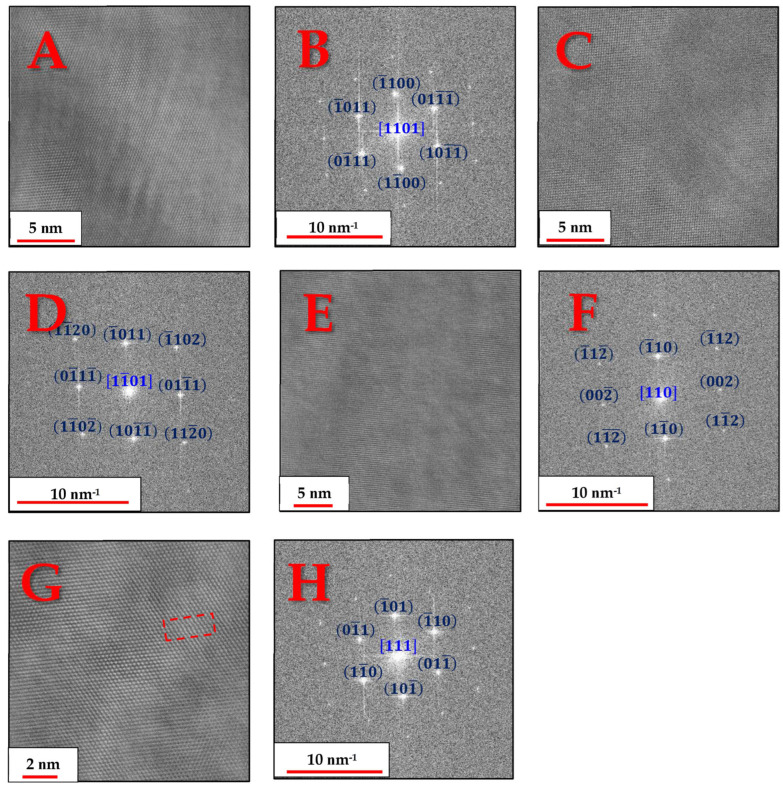
(**A**,**C**) Radial Wiener-filtered STEM-HAADF micrographs showing lattice images from zone axes $\left[1101\right]$ and $\left[1\bar{1}01\right]$, respectively, of α-Ti grains and (**B**,**D**) their corresponding resolved FFT patterns (space group 194, $P6_{3}/mmc$); (**E**,**G**) Radial Wiener-filtered STEM-HAADF micrographs showing lattice images from zone axes $\left[110\right]$ and $\left[111\right]$, respectively, of β-Ti grains and (**F**,**H**) their corresponding resolved FFT patterns (space group 229, $Im\bar{3}m$).

The typical composition of the individual phases (as an average of multiple sampling) is provided in Table 3:

In order to verify the existence of the α and β phases, several grains of both phases were aligned in a zone axis and the corresponding lattice images and FFT patterns were obtained, as shown in Figure 7:

The above lattice images and FFT patterns are indicative of the hexagonal structure of the α-Ti grains and the cubic (BCC) structure of the β-Ti particles. The FFT patterns in Figure 7F,H indicate that there may be significant deviations from equilibrium interplanar distances typical of BCC titanium crystals. In the $\left[110\right]$ zone axis image (Figure 7E), the angle between the $\left(002\right)$ and $\left(1\bar{1}0\right)$ atomic planes is found to be 92.5° instead of 90°, while in the $\left[111\right]$ zone axis image (Figure 7G), the inter-planar distances of one of the $\left\{110\right\}$ planes is found to be significantly shorter than the other two.

This deviation from equilibrium distances and angles is most likely associated with residual strains that could not be fully relieved during the heat treatment. The residual strains are caused by the coherent precipitation of the β phase from its α′ martensite predecessor. Since at 800 °C, the diffusion of Ti and other alloying elements is still sluggish, this can also lead to a mismatch between atomic layers in the precipitating β phase, possibly even forming dislocations, as can be seen in the dashed red rectangle in the dashed red rectangle in Figure 7G. Although the mechanical properties were not investigated in this publication, this coherent bonding of the α and β phases most likely leads to increased strength and reduced ductility compared to incoherent bonding, due to these interfaces serving as excellent barriers to dislocation movement [23].

**Figure 8:**
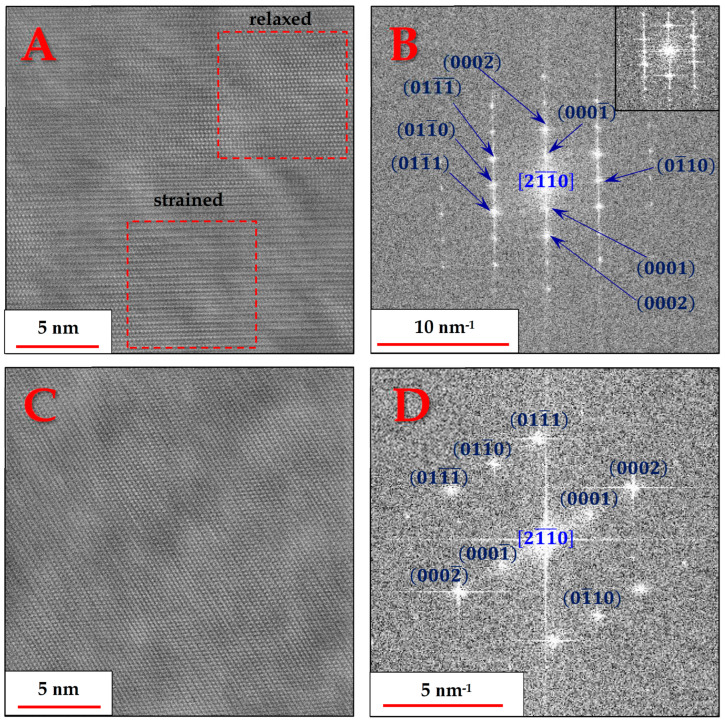
Radial Wiener-filtered STEM-HAADF micrographs showing the lattice images (**A**,**C**), and the corresponding resolved FFT patterns (**B**,**D**) of two α-Ti grains, both oriented in the $\left[2\bar{1}\bar{1}0\right]$ zone axis. The inset in B shows the FFT of the relaxed region only, which lacks the $\left(0001\right)$ reflections.

Up to this point, the nano-features could not be observed and identified, and it became clear that a clue might be found in α-Ti grains that could be aligned in one of the central zone axes of the HCP structure. Figure 8 demonstrates the recurring anomaly found in α-Ti grains in the form of high-order periodicity, pertaining to the normally forbidden reflections of the $\left(0001\right)$ planes [24], when viewed from the zone axis $\left[2\bar{1}\bar{1}0\right]$:

This atomic ordering anomaly, caused by the high residual strains found in the α-Ti phase, manifested itself, in varying severity degrees, as unequal brightness in successive $\left(0002\right)$ planes. In other words, every other $\left(0002\right)$ plane experiences some form of strain, causing its atomic rows to shift ever so slightly from their equilibrium position. Figure 7A shows that while some areas of the α grains appear normal or relaxed, i.e., with only their $\left(0002\right)$ planes being periodic, other areas, just a few nanometers away, are completely strained.

**Figure 9:**
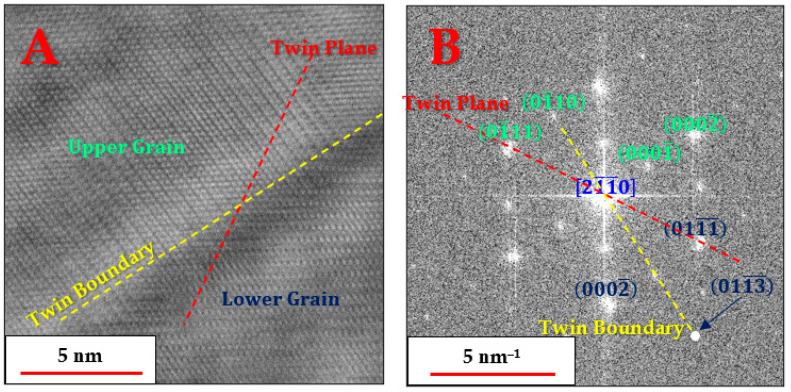
Radial Wiener filtered STEM-HAADF micrograph showing the lattice image (**A**) and the corresponding resolved FFT (**B**) of two twinned α-Ti grains with a shared $\left[2\bar{1}\bar{1}0\right]$ zone axis and twin plane of the $\left\{01\bar{1}\bar{1}\right\}$ plane family.

The residual strains persisting in the alloy, and specifically in the α-Ti phase, were also present in the form of twinning. Figure 9 shows one such instance where two α-Ti grains oriented in the $\left[2\bar{1}\bar{1}0\right]$ zone axis have a twin plane that is different from the boundary plane:

It is noticeable that the twin boundary is different in each of the two grains. For the lower grain, it is the $\left(01\bar{1}\bar{3}\right)$ plane, while for the upper grain, it is the $\left(01\bar{1}0\right)$ plane. The formation of twins serves as another indication of the heavy strains developing in the alloy as the β phase starts precipitating.

Thus, it can be said that the precipitation of the β phase does not reach the maturation stage after heat-treating at 800 °C. In other words, this temperature may not be ideal since it does not enable the growth of the β phase to the extent that all stresses arising from its coherence with the neighboring α phase can be relieved. “Mature” β precipitation occurs at higher temperatures as attested to by micrographs taken at higher temperatures, which do not typically exhibit the nano-features in the α phase [2,6,21]. Although this topic is not covered in this publication, another aspect to bear in mind when analyzing the nano-features is their influence on the overall mechanical properties. Usually, nanoparticles increase yield and ultimate tensile strengths while decreasing the overall ductility. However, when the “nanoparticles” essentially comprise the same phase and the only markers serving as the boundary between them and the surrounding phase are strains resulting from lattice distortion, the contribution to the overall mechanical properties may be small. O. Dolev et al. [25] provide thorough research on the fatigue and fracture toughness of AM Ti-6Al-4V products following an identical 800 °C tempering treatment that may shed some light on a possible link between the nano-scale features and the mechanical properties.

Literature evidence for heavy straining in HCP metals can be found in the work of Wang et al. for Mg (Figure 2C in [26]). They provide evidence for the associated twinning formation, which manifests itself as a high-order periodicity displaying the forbidden (0001) plane reflections when viewed from either of the $\langle 2\bar{1}\bar{1}0\rangle$ zone axes. Having said that, the straining phenomenon in [26] was caused by compression, whereas in the current investigation, the straining is a result of β-Ti phase precipitation during heat treatment.

The fact that the only anomaly observed inside α-Ti grains was the periodicity of the $\left(0001\right)$ planes in their HCP crystal structure proves that the nano-features can be neither a variant of the α phase with a different spatial orientation nor a different crystal structure pertaining to some other phase, such as α″. In light of the above, what is the nature of the nano-sized features easily observed in HR-SEM analysis? To answer this question, one more lattice image had to be obtained, showing the boundary between the precipitating β-phase and the α phase.

**Figure 10:**
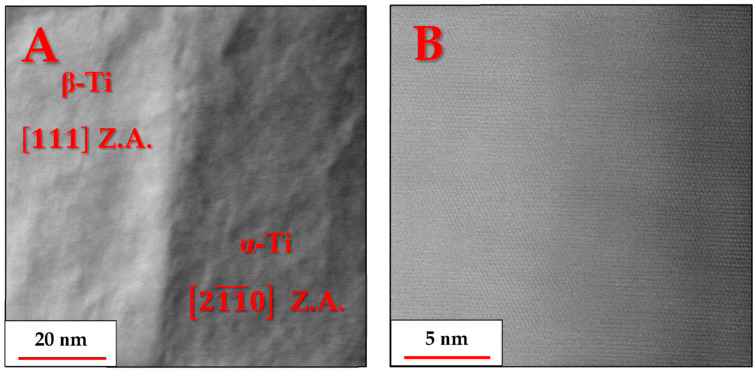
Radial Wiener-filtered STEM-HAADF micrographs at (**A**) low and (**B**) high magnifications respectively, showing the lattice image of the boundary region between adjacent β-Ti and α-Ti grains, each oriented in its respective zone axis.

The α/β interface regions exhibited coherence, as demonstrated by the smooth transition between the atomic planes of both phases. One such example is provided in Figure 10, with an α/β orientation relationship of ${\left[111\right]}_{\beta }{\left(1\bar{1}0\right)}_{\beta }||{\left[2\bar{1}\bar{1}0\right]}_{\alpha }{\left(0001\right)}_{\alpha }$, which is in agreement with the well-known orientation relationships of α + β titanium alloys [13].

When combining the HRTEM findings with the HRSEM micrographs, it can be inferred that a large fraction of the volume of each α-Ti grain, especially near the boundaries with the β-Ti phase particles, as shown in Figure 9, consists of the strained version of the α-Ti phase, displaying the forbidden (0001) plane reflections. In contrast, the nano-sized features are probably simply the relaxed version of the α-Ti phase, as also captured in Figure 8A, which would be expected to be less susceptible to etching in Kroll’s reagent than the surrounding strained matrix and thus would protrude more when viewed in HRSEM.

In more detail, the clear boundaries between the nano-features and the surrounding α-Ti grain matrix are only observable when the metallographic specimen is etched, due to the fact that lattice distortion and internal stresses significantly increase etching rates. Otherwise, the nano-features are indistinguishable, both in HR-SEM or HR-S/TEM, from their surroundings. That is, of course, because they have the exact same composition and crystal orientation as their surrounding matrix grain. All the relaxed α-Ti domains share the same nano-sized scale; however, they are not identical in morphology. More specifically, some grains show more elongated nano-features, which is most probably because the entire matrix grain is oriented such that the anisotropic dimension of the unit cell, the c lattice parameter, is almost perfectly perpendicular to the sample surface normal.

The scalability of our microstructural findings to larger printed Ti-6Al-4V parts will be determined by how the heat transfer regime changes. This regime is influenced by various printing parameters (e.g., the laser power, scanning speed, hatch distance and layer thickness) and different heat treatment conditions. Nevertheless, the fundamental microstructure comprising α-Ti and β-Ti phases, along with both relaxed and strained forms of α-Ti, is expected to persist in larger parts, albeit with potentially different heat treatment temperature ranges and particle or domains sizes [27,28].

## 4. Conclusions

The heat treatment of additively manufactured Ti-6Al-4V performed at 800 °C results in the decomposition of the α′ martensite into α lamellae and β particles situated mostly at α grain boundaries. Both the α and β phases are heavily strained as a result of the α′ decomposition process. The straining of the α-Ti grains is non-uniform, with alternating nano-sized islands of relaxed regions inside a largely strained form of the HCP crystal structure. These relaxed regions appear as nano-sized particles or features under a scanning electron microscope when an etched metallographic specimen is used.

The relaxed nano-sized regions of the α-Ti phase exhibited an average characteristic size of 69.6 mm and a median of 54.8 nm. Their volume fraction was determined to be 17.21%.

The authors’ hypothesis is that the 800 °C thermal treatment does not enable the as-built all-martensitic structure to fully mature into an α + β microstructure, resulting in heavy internal stresses and lattice distortions due to coherent phase boundaries forming between both Ti allotropes. These, in turn, would be expected to lead to increased yield and ultimate tensile strength values, while reducing the overall elongation or plasticity, compared to a wrought Ti-6Al-4V alloy.

## Figures and Tables

**Table 1:** Chemical composition of raw material Ti-6l-4V powder and final AM product.

Element	H	C	N	O	Al	V	Fe	Ti
Powder Sample	0.002	0.012	0.011	0.14	6.41	4.10	0.19	Balance
Final Product	Non-detectable	0.016	0.018	0.14	6.28	4.15	0.19	Balance

**Table 2:** Evaluated fraction of each phase or feature in AM Ti-6Al-4V microstructure following heat treatment at 800 °C:

Phase/Feature	Volume Fraction [%]
β-Ti	9.33
α-Ti (relaxed nano-features)	17.21
α-Ti (strained matrix grain)	73.46

**Table 3:** EDX area measurements of single phases in atomic and weight fractions.

Phase	Atomic Fractions			
	Al [%-at.]	V [%-at.]	Fe [%-at.]	Ti [%-at.]
α	11.0%	2.9%	0.5%	85.6%
β	3.5%	24.3%	3.1%	69.1%
	**Weight Fractions**			
α	6.5%	3.2%	0.6%	89.7%
β	1.9%	25.8%	3.5%	68.8%

## Data Availability

The raw data supporting the conclusions of this article will be made available by the authors on request.
